# Predictors of pregnancy stress and psychological birth trauma in women undergoing vaginal delivery: a cross-sectional study in China

**DOI:** 10.1186/s12884-023-05890-1

**Published:** 2023-08-22

**Authors:** Dongmei Ma, Shiwen Sun, Jialu Qian, Man Wang, Huimin Gu, Jingjing Lou, Xiaoyan Yu

**Affiliations:** 1 Department of nursing, Women’s Hospital School of Medicine, Zhejiang University, Hangzhou, Zhejiang China; 2 Zhejiang University School of Medicine, Hangzhou, Zhejiang China

**Keywords:** Psychological birth trauma, Postpartum posttraumatic stress disorder, Pregnancy stress, Social support, Family support

## Abstract

**Background:**

Psychological birth trauma exhibits a high incidence worldwide, resulting in a wide range of negative impacts on mothers, infants, couples, families and society at large through the maternal-centered ripple effect. However, there is currently limited research on psychological birth trauma in China. Social support and pregnancy stress are important influencing factors of psychological birth trauma. Consequently, this study aimed to explore predictors of pregnancy stress and psychological birth trauma in women undergoing vaginal delivery in China.

**Methods:**

This cross-sectional study was performed at a single medical center between December 2021 and May 2022 in Hangzhou, China. Participants were selected using a convenience sampling technique. A total of 351 postpartum women within one week after vaginal delivery were included. Questionnaires were used to collect sociodemographic and obstetric characteristics and scores on the Pregnancy Stress Rating Scale (PSRS), City Birth Trauma Scale (City BiTS), Social Support Rating Scale (SSRS) and Family Adaptation Partnership Growth Affection and Resolve index (Family APGAR). Both univariate analysis and multiple linear regression analysis were conducted to assess predictors of pregnancy stress and psychological birth trauma.

**Results:**

The median (IQR) of PSRS and City BiTS scores were 10.00 (14.00) and 3.00 (9.00), respectively. The incidence of postpartum posttraumatic stress disorder was 4.0% (14/351). Parity, social support, family support and level of education were predictors of pregnancy stress. Delivery complications, psychological traumatic event, pregnancy stress and family support were predictors of psychological birth trauma (P < 0.05).

**Conclusion:**

Pregnancy stress is related to social support, family support and some sociodemographic and obstetric characteristics. Psychological birth trauma is correlated with delivery complications, psychological traumatic event, pregnancy stress and family support. Consequently, enhancing social support, especially family support, for pregnant women as a means of reducing pregnancy stress can effectively prevent psychological birth trauma.

## Background

Psychological birth trauma (PBT) refers to the actual or threatening serious injury or even death caused to the parturient, fetus or newborn during pregnancy and delivery, which makes the parturient experience a series of psychological pain and negative psychological reactions, such as strong fear, helplessness and hopelessness [1]. If severe PBT symptoms are not controlled, they may evolve into postpartum posttraumatic stress disorder (PPTSD), resulting in symptoms of intrusive memories, negative cognition, avoidance behavior, sustained increased alertness and other PPTSD symptoms [2]. At present, there is no unified diagnostic standard for PBT at home or abroad, and the incidence of PBT varies in different countries and regions. Approximately 9.1–57.2% of women underwent traumatic birth experiences, and about 1.2–20% of women developed PPTSD [3–6].

Beck used the “ripple effect” to describe the harm of PBT [7]. PBT can have serious and lasting impacts on the mother, baby, family, and medical staff. For the parturients themselves, PBT may directly cause anxiety, depression, fear and other psychological pain [8–10]. In severe cases, parturients may experience nightmares, relive the delivery experience, or forget delivery memories [2, 11]. These symptoms can affect victim’s daily lives, and some may attempt to avoid subsequent pregnancies or refuse to give birth again [12]. Traumatic births may cause mothers to stop breastfeeding prematurely or to refuse to breastfeed, leading to lower rates of breastfeeding [13] and endangering the mother-child relationship. Birth trauma may also affect the relationship between the mother and other family members, particularly relationships with partners [14].

Many factors impact PBT, including objective factors like severe labor pain, lack of prenatal health education or delivery guidance, unmet delivery expectations, and unfamiliar delivery environment [9, 10, 15, 16], as well as subjective factors, such as poor communication between doctors and patients, feeling forgotten and ignored, worries about the health or life of the infant, insufficient family support and/or social support, and mental health during pregnancy, etc. [9, 15–19]. One study reported that pregnancy stress is positively correlated with PPTSD, whereas social support is negatively correlated with PPTSD. Thus, pregnancy stress is the mediating variable between maternal PPTSD and social support [19].

In recent years, PBT has attracted the attention of scholars at home and abroad. However, existing studies primarily focus on the secondary psychological problems caused by PBT, such as postpartum depression and PPTSD, and rarely focus on PBT itself [20]. In addition, due to the lack of specific assessment tools, most studies on PBT are qualitative studies of emotional experience [21, 22]. The few quantitative surveys primarily rely on the use of a PPTSD-related scale to determine the PBT. Therefore, PBT studies in the Chinese cultural context can help the design and identification of accurate and effective psychological intervention programs. In this cross-sectional study, we sought to determine the predictors of PBT immediately after vaginal delivery, particularly focusing on the influence of pregnancy stress, family support and social support. In addition, we also explored predictors of pregnancy stress.

## Methods

### Study design and participants

A cross-sectional descriptive study was performed in a third-level A-grade obstetrics and gynaecology hospital from December 2021 to May 2022. Convenience sampling was used to recruit women following vaginal delivery. The inclusion criteria were as follows: (1) age greater than 18 years, (2) status post vaginal delivery, (3) 0 to 7 days postpartum, (4) ability to communicate and understand the questionnaire, and (5) voluntary participation in the study and completion of informed consent. The exclusion criteria included the following: (1) severe postpartum complications and inability to participate in the questionnaire and (2) concurrent major physical or mental health disorder. The sample size estimation method for studies exploring predictive factors indicated that a sample size of at least 5–10 folds the number of variables was necessary [23], therefore, we estimated an sample size of 150–300 for this study.

### Data collection

This study was approved by the ethics committee of the Women’s Hospital School of Medicine, Zhejiang University (IRB No. PRO2021-1865). A pilot study was performed with 15 eligible subjects to evaluate the feasibility of the study and to identify any unsuitable items. All data were collected by well-trained collectors using a face-to-face data collection method and were confidential. Following a script, the researchers explained the purpose and significance of the study and the procedure for filling in the questionnaire to each eligible subjects during their postpartum hospitalization period. Participants filled out electronic questionnaires after providing informed consent. The researchers further checked the completeness of the questionnaire on a daily basis. 380 participant charts were reviewed prior to ascertaining eligibility, of these, 360 participants met the inclusion criteria, 351 participants completed questionnaires, representing a response rate of 97.5%, the remaining participants refused to participate in research or did not complete all questionnaire items due to lack of time or unwillingness to participate.

### Instruments

In this study, sociodemographic and obstetric characteristics questionnaires, the Pregnancy Stress Rating Scale (PSRS), City Birth Trauma Scale (City BiTS), Social Support Rating Scale (SSRS) and Family Adaptation Partnership Growth Affection and Resolve index (Family APGAR) were used to collect data.

The sociodemographic and obstetric characteristics questionnaires were created based on the influencing factors of PBT in previous studies and the researchers’ clinical work experience, which comprised two parts as follows: sociodemographic characteristics (including age, level of education, residential area, income level, employment status, and religious beliefs) and obstetric characteristics (including pregnancy intention, manner of fertilization, abnormal pregnancy history, parity, gestational age at delivery, pregnancy complications, delivery complications, perineal laceration degree, newborn abnormalities, location of the newborn, degree of labor pain, analgesic methods of labor pain, analgesic methods of postpartum pain, care experience during childbirth, neglect during childbirth, loss of dignity during childbirth, privacy disclosure during childbirth, and psychological traumatic events of childbirth).

### PSRS

The Pregnancy Stress Rating Scale (PSRS) was developed by Chen, a Chinese scholar from Taiwan, in 1983 [24]. The scale includes three dimensions, including stress caused by worrying about the health and safety of the mother and child, stress caused by recognizing the role of the mother, and stress caused by worrying about changes in body shape and body function, with a total of 30 items. Each item is scored 0–3 points. Higher total scores represent more significant pregnancy stress. The Cronbach’s α of the scale was 0.90 [25].

### City BiTS

The City Birth Trauma Scale (City BiTS) was formulated by British scholar Ayers et al. [26] in 2018 according to the latest diagnostic criteria of PTSD in the DSM-5 and was translated into Chinese and tested for reliability and validity by Shen et al. [27]. The scale is a specific assessment tool used to measure PPTSD related to childbirth. The scale contains 29 items that are divided into two dimensions: birth-related symptoms and general symptoms. PTSD is evaluated according to whether the patient has experienced traumatic events (Items 1–2), the frequency of related symptoms (Items 3–22), whether there are separation symptoms (Items 23–24), symptom occurrence time (item 25), the duration of symptoms (Item 26), whether there is pain and social dysfunction (Items 27–28), and whether the symptoms are related to drugs (Item 29). The total score of the frequency of related symptoms is 0 ~ 60, which we used to measure PBT in this study. Cronbach’s α of the scale is 0.934 [27].

### SSRS

The Chinese version of the self-reported Social Support Rating Scale (SSRS) was used to evaluate maternal social support. This scale was created by Xiao in 1986 [28] and modified and improved in 1990. The SSRS has been widely used to assess the social support levels of various populations and includes the following three subscales: objective support (Items 2, 6, and 7), subjective support (Items 1, 3, 4, and 5), and availability of support (Items 8–10). Higher total scores reflect greater social support. Total scores < 35, 35–45, and > 45 indicate low, moderate, and strong social support, respectively. The scale exhibited good reliability and validity, with a retest reliability r of 0.92 and Cronbach’s α of 0.89–0.94 [29].

### Family APGAR

The Family Adaptation Partnership Growth Affection and Resolve index (Family APGAR) was designed by Smilkstein G in 1978 [30] and is primarily used to evaluate patient satisfaction with aspects of family functioning: adaptation, partnership, growth, affection and resolve. This scale is a five-item, three-point scale ranging from 0 (hardly never) to 2 (always). Total scores ≥ 7, 4–6, and 0–3 represent good family function, moderate obstacles in family function, and serious obstacles in family function, respectively. Cronbach’s α of the scale is 0.89 [31].

### Data analysis

All data were analyzed using SPSS 20.0 and crosschecked for verification. Continuous variables were presented as the mean ± standard deviation (SD) or median and interquartile range (IQR) when appropriate. Correlations were evaluated using Spearman’s correlation analyses, and univariate analyses were conducted using t tests, one-way ANOVA, the Mann–Whitney test or Kruskal–Wallis test depending on the type of data. A multivariate linear regression analysis was performed to identify predictors of pregnancy stress and PBT. *P* values < 0.05 were considered statistically significant.

## Results

### Participant characteristics

A total of 351 postpartum women participated and completed all of the questionnaires. The ages of the women ranged from 22 to 43 years (median = 30.00, IQR = 5.00), and their gestational age at delivery ranged from 26 to 41 weeks (median = 39.00, IQR = 2.00). Among them, 259 (73.8%) women were primiparous, and 92 (26.2%) were multiparous. The remaining sociodemographic and obstetric characteristics are presented in Table 1.

**Table 1:** Characteristics of participants and their relationship with PSRS and City BiTS (*n* = 351)

**Characteristics**	**Number (%)**	PSRS	*P* value	City BiTS	*P* value
		Mean (SD)		Mean (SD)	
Age			0.011^b^		0.308^b^
21–25	27 (7.7)	7.67 (6.80)		3.67(4.62)	
26–30	157(44.7)	12.83 (10.38)		5.85(7.71)	
31–35	136(38.7)	12.88 (11.08)		5.96(6.94)	
≥35	31(8.8)	7.94 (6.98)		3.48(3.92)	
Education level			0.037^b^		0.976^b^
Junior high school/below	11(3.1)	11.00 (9.06)		4.73(5.73)	
Senior high school	28(8.0)	10.00 (9.73)		5.18(6.00)	
Junior college	65(18.5)	10.14 (11.70)		5.09(6.02)	
Bachelor’s degree/above	247(70.4)	12.79 (10.01)		5.70(7.38)	
Residential area			0.752^b^		0.899^b^
City	292(83.2)	11.86 (9.75)		5.51(7.13)	
Towns	30(8.5)	10.80 (9.47)		5.43(5.58)	
Villages	29(8.3)	14.83 (15.55)		5.66(6.99)	
Level of income			0.074^a^		0.530^a^
High	196(55.8)	11.11 (9.77)		5.16(6.95)	
Medium	155(44.2)	13.17 (10.91)		5.96(7.02)	
Employment status			0.192^a^		0.705^a^
Employed	283(80.6)	12.23 (9.98)		5.66(7.30)	
Unemployed	68(19.4)	11.13 (11.69)		4.90(5.47)	
Religious belief			0.673^a^		0.884^a^
No belief	340(96.9)	12.05 (10.33)		5.52(7.02)	
Had beliefs	11(3.1)	11.09 (10.52)		5.27(5.90)	
Pregnancy intention			0.224^a^		0.747^a^
Planned pregnancy	288(82.1)	12.30 (10.23)		5.56(7.02)	
Unplanned pregnancy	63(17.9)	10.75 (10.72)		5.33(6.85)	
Manner of fertilization			0.242^a^		0.772^a^
Conceived naturally	322(91.7)	11.88 (10.36)		5.61(7.11)	
Assisted reproduction	29(8.3)	13.59 (10.00)		4.48(5.40)	
Abnormal pregnancy history			0.789^a^		0.279^a^
Never experienced	277(78.9)	11.98 (10.45)		5.33(7.01)	
Had experienced	74(21.1)	12.16 (9.89)		6.20(6.90)	
Parity			0.001^a^		0.343^a^
Primiparous	259(73.8)	13.11 (10.79)		5.76(7.33)	
Multiparous	92(26.2)	8.95 (8.18)		4.82(5.89)	
Gestational age at delivery			0.528^a^		0.402^a^
< 37 weeks	22(6.3)	9.86 (7.10)		4.73(7.43)	
≥37weeks	329(93.7)	12.16 (10.50)		5.57(6.96)	
Pregnancy complications			0.283^a^		0.352^a^
Absent	250(71.2)	11.73 (10.48)		5.41(7.14)	
Present	101(28.8)	12.72 (9.94)		5.78(6.59)	

a Mann−Whitney U test

b Kruskal Wallis test

### Pregnancy stress, psychological birth trauma, social support and family support

The median (IQR) of PSRS and City BiTS scores were 10.00 (14.00) and 3.00 (9.00), respectively. A total of 23.9% (84/351) of parturients experienced childbirth trauma. The incidence of PPTSD was 4.0% (14/351). The median (IQR) of SSRS score was 44.00 (9.00), and the proportions of strong, moderate and poor social support were 41.3% (145/351), 47.0% (165/351) and 11.7% (41/351), respectively. The median (IQR) of Family APGAR score was 10.00 (3.00). Based on the abovementioned criteria, 19.9% of the women exhibited serious or moderate obstacles in family function, and 80.1% had good family function.

### Bivariate association between pregnancy stress or psychological birth trauma and sociodemographic and obstetric characteristics

To avoid omission of factors that might fail to exhibit significance in univariate analyses but could exhibit significance in multifactor analysis, P ≤ 0.1 was considered statistically significant in the univariate analysis. Tables 1 and 2 present the bivariate associations between the study variables and PSRS or City BiTS. Univariate analyses indicated that four factors were associated with PSRS: age, level of education, income level and parity. Additionally, six factors were associated with City BiTS: delivery complications, degree of labor pain, analgesic methods of postpartum pain, loss of dignity during childbirth, privacy disclosure during childbirth, and psychological traumatic events of childbirth.

**Table 2:** Delivery related factors of participants and their relationship with City BiTS (*n* = 351)

Delivery related factors	Number (%)	City BiTS	*P* value
		Mean (SD)	
Delivery complications			0.001^a^
Absent	282(80.3)	5.03(6.39)	
Present	69(19.7)	7.51(8.77)	
Perineal laceration degree			0.351^b^
Intact perineum	34(9.7)	4.50(7.00)	
Minor laceration	211(60.1)	5.65(6.43)	
Episiotomy	94(26.8)	5.02(6.20)	
Serious laceration	12(3.4)	9.92(16.20)	
Newborns abnormalities			0.223^a^
Absent	289(82.3)	5.16(6.25)	
Present	62(17.7)	7.19(9.59)	
Location of the newborn			0.511^a^
Room of mother and infant	249(70.9)	5.44(6.37)	
Neonatology department	102(29.1)	5.70(8.33)	
Degree of labor pain			0.075^b^
Slight pain	9(2.6)	4.78(7.93)	
Moderate pain	107(30.5)	4.74(6.43)	
Intense pain	235(67.0)	5.90(7.18)	
Analgesic methods of labor pain			0.449^b^
Labor analgesia	248(70.7)	5.76(7.52)	
Non-drug analgesia	53(15.1)	4.53(4.63)	
Other methods	7(2.0)	10.14(7.9)	
Absent	43(12.3)	4.56(5.65)	
Analgesic methods of postpartum pain			0.021^b^
Analgesic drugs	97(27.6)	6.89(6.88)	
Non-drug analgesia	62(17.7)	4.60(5.36)	
Other methods	9(2.6)	2.56(3.40)	
Absent	183(52.1)	5.25(7.54)	
Care experience during childbirth			0.240^a^
Good	324(92.3)	5.46(7.04)	
Common	27(7.7)	6.22(6.30)	
Neglected during childbirth			0.273^a^
Absent	328(93.4)	5.27(6.34)	
Present	23(6.6)	8.96(12.86)	
Loss of dignity during childbirth			0.039^a^
Absent	334(95.2)	5.28(6.46)	
Present	17(4.8)	10.18(13.15)	
Privacy disclosure during childbirth			0.070^a^
Absent	312(88.9)	5.24(6.49)	
Present	39(11.1)	7.72(9.94)	
Psychological traumatic events			0.001^a^
Absent	341(97.2)	5.18(6.30)	
Present	10(2.8)	16.90(15.77)	

a Mann−Whitney U test

b Kruskal Wallis test

### Correlations among PSRS, SSRS, Family APGAR and City BiTS

Correlation analyses revealed that PSRS was negatively correlated with SSRS and Family APGAR to a moderate degree. Similarly, City BiTS exhibited a low negative correlation with SSRS and Family APGAR but a moderate positive correlation with PSRS. Table 3 presents the associations between the scores of all SSRS dimensions and PSRS and City BiTS scores.

**Table 3:** The correlation among PSRS, City BiTS, SSRS and Family APGAR

	PSRS		City BiTS	
	r	p	r	p
PSRS	-	-	0.442	<0.001
SSRS	-0.258	<0.001	-0.199	<0.001
Objective support	0.000	0.996	0.011	0.834
Subjective support	-0.358	<0.001	-0.273	<0.001
Availability of support	-0.099	0.063	-0.154	0.004
Family APGAR	-0.282	<0.001	-0.207	<0.001

### Multifactorial analysis of pregnancy stress and psychological birth trauma

Multivariate linear regression analyses were conducted with PSRS and City BiTS as the dependent variables and the aforementioned significantly associated factors as independent variables. There was no multicollinearity among the independent variables in the two regression models (VIF<5). The results suggest that parity, social support, and family support were associated with decreased risks of pregnancy stress. In contrast, level of education was associated with an increased risk of pregnancy stress (see Table 4). With respect to City BiTS, delivery complications, psychological traumatic event, and pregnancy stress were important risk factors that could significantly increase PBT, whereas family support was a protective factor against PBT (see Table 5).

**Table 4:** Multivariate linear regression analyses predicting pregnancy stress(n = 351)

Variable	B	Beta	t	*P*	B 95% CI	
					Lower	Upper
Constant	28.735	--	7.040	<0.001		
Education level	1.867	0.140	2.747	0.006	0.530	3.204
Parity	-3.785	-0.161	-3.158	0.002	-6.143	-1.427
Social support	-0.239	-0.166	-3.046	0.002	-0.394	-0.085
Family support	-0.959	-0.190	-3.496	0.001	-1.498	-0.419

Adjusted R^2^ = 0.115, F = 12.356, P<0.001

**Table 5:** Multivariate linear regression analyses predicting psychological birth trauma(n = 351)

Variable	B	Beta	t	*P*	B 95% CI	
					Lower	Upper
Constant	-6.475	-	-2.351	0.019	-11.893	-1.058
Complications during delivery	2.060	0.117	2.509	0.013	0.445	3.675
Psychological traumatic event	9.678	0.231	4.929	P<0.001	5.816	13.540
Pregnancy stress	0.242	0.358	7.451	P<0.001	0.178	0.306
Family support	-0.390	-0.114	-2.376	0.018	-0.713	-0.067

Adjusted R^2^ = 0.246, F = 29.531, P<0.001

## Discussion

In the present study, the median (IQR) of PSRS scores were 10.00 (14.00), significantly lower than that in a previous study [32]. The difference may be due to different study populations and data collection times. Our study included women who were one week postpartum after vaginal delivery. Thus, the women may have been more relaxed when filling out the questionnaires. The questionnaire was primarily completed by recalling stress during pregnancy; therefore, the total stress score may underestimate the true stress during pregnancy.

PBT has a wide and far-reaching impact on women. Medical staff should therefore improve the evaluation and management of PBT in women. This study focused on the PBT immediately within one week after vaginal delivery, whereas previous studies focused on PPTSD, typically through evaluation several months after delivery. The present study revealed the median (IQR) of City BiTS scores was 3.00 (9.00), and the incidence of PPTSD was 4.0%, lower than that in previous studies [6, 27, 33], similar to that in a systematic review [34]. These results may differ due to differences in assessment tools and data collection times. Furthermore, this study only included women who gave birth vaginally, other studies included women who gave birth by cesarean section, and caesarean section especially emergency cesarean section is an independent risk factor for PPTSD [27]. This study showed that PBT was not solely related to a cesarean birth, some women who delivered vaginally also experienced PBT.

Our results suggested that parity was associated with a decreased risk of pregnancy stress, consistent with previous studies [32, 35]. A possible explanation for this may be that nulliparous women have not previously undergo pregnancy and are therefore concerned due to inexperience, resulting in increased pregnancy stress. Multiparous women, by contrast, have practical experience; therefore, their level of stress is less than that of nulliparous women [35]. Thus, medical staff should provide more guidance to primiparous women during pregnancy, improve their understanding of pregnancy and delivery, reduce pregnancy stress caused by lack of relevant knowledge, and help these women better navigate pregnancy and delivery [36].

Our results suggested that social and family support were associated with decreased risks of pregnancy stress, which is consistent with other studies [19, 37]. Good social support can improve pregnant women’s understanding of stressful events, allowing them to adapt to their role as mothers as soon as possible. The family is an important source of social support for pregnant women. The support of family members, especially spouses, can alleviate the adverse effects of stressful events on pregnant women, enhance their self-confidence, increase their emotional support, and thus reduce pregnancy stress [38]. Therefore, medical staff should actively mobilize the maternal social support system, encourage family members, especially spouses, to participate in prenatal care, strengthen care and spiritual support for pregnant women, be cognizant of psychological changes of pregnancy, create a warm and happy family environment for pregnant women, and reduce pregnancy stress to the greatest extent [38]. In addition, medical staff should also guide pregnant women to reasonably use their social support system to deal with stressful pregnancy events.

In contrast, level of education was associated with an increased risk of pregnancy stress, consistent with the results of studies conducted by Bao et al. [39]. Pregnant women with high education levels may also experience greater work stress. Additionally, the greater access to social information from various sources may increase the likelihood that they receive bad information. Therefore, these women are more likely to experience negative emotions, such as tension and anxiety, during pregnancy. For the above groups, medical staff should provide comprehensive and scientific guidance during pregnancy and correct women’s misconceptions in a timely manner.

Our study indicated that delivery complications were important risk factors that could significantly increase PBT. Previous studies also reported that poor health and pregnancy complications were related to PPTSD [6, 40]. Delivery complications can cause physical harm to mothers and babies, and mothers may experience greater pain or physical discomfort, which can lead to increased PBT [33].

Psychological trauma events, such as severe labor pain, distressing labor experience, witnessing the childbirth of other women, premature delivery, and the separation of mothers and infants, were important risk factors that significantly increased PBT. This finding is consistent with a previous qualitative study investigating traumatic childbirth experiences [15, 17, 41]. The study by Ayers et al. indicated that the most strongly related risk factor for PTSD was negative subjective birth experiences [40]. Women who experience psychological trauma events may have negative subjective birth experiences and increased emotional birth trauma [17], thereby increasing PBT. Therefore, measures should be taken to reduce labor pain and improve the delivery environment, such as setting up family-integrated delivery rooms or increasing the number of single delivery rooms to improve the delivery experience. Strengthening prenatal education to reduce maternal anxiety and fear of delivery, so as to reduce the level of PBT. Additionally, it is necessary to discuss with all pregnant women their expectations for pregnancy and birth before delivery, and then ask them if everything is as they expected after delivery. If not, these women need special attention from healthcare providers to prevent the occurrence of PPTSD.

In the current study, pregnancy stress was an important risk factor that could significantly increase PBT, whereas family support was a protective factor against PBT. This finding is consistent with previous research results [17–19, 33]. Pregnant women experiencing greater stress during pregnancy may exhibit more negative emotions, such as tension and anxiety, which tend to become increasingly strong as they go through childbirth [10]. Furthermore, women with high levels of stress during pregnancy tend to be women with anxiety traits, which is a risk factor for negative delivery experiences, and these women are more likely to regard childbirth as traumatic [42]. In addition, pregnant women with high pregnancy stress often suffer from low social support. Additionally, when pregnant women are under high stress, they cannot fully perceive and access social support [43] and are more likely to suffer from PPTSD. Good social support can promote regulation of negative emotions and positive coping. As an important source of social support, family members, especially spouses, are a protective factor for women’s mental health throughout the perinatal period, which can effectively alleviate the adverse effects of stressful events and reduce negative perceptions during delivery. Therefore, medical staff should evaluate maternal pregnancy stress, social support and perinatal PTSD and provide personalized suggestions as necessary. We should not only strengthen medical staff support of women but also strengthen family support and encourage spouses to actively participate in prenatal care, thereby assuring that pregnant women can receive more attention and decreasing the incidence of PBT. Furthermore, we should aid pregnant women in effectively accessing social support to reduce pregnancy stress, thereby decreasing the risk of PBT and improving maternal and neonatal delivery outcomes [19].

### Strengths and limitations

This study randomly selected one hospital in Hangzhou to investigate predictors of pregnancy stress and PBT in women status post vaginal delivery, which will help fill an important gap and raise awareness of this issue. Several limitations of this study should be noted. First, the postpartum women completed the PSRS by reviewing pregnancy stress; thus, we cannot exclude the possibility of recall bias. Second, as there is no special scale to measure PBT, this study used the City BiTS, which is primarily used to measure PPTSD. Third, due to its cross-sectional nature, one of the limitations of the current study is that the relationships shown between PBT and pregnancy stress and social support and some sociodemographic characteristics cannot accurately indicate a causal relationship. In addition, the non-probabilistic convenience sampling method used may limit the generalizability of the findings, and the study population was small and recruited from a single hospital, potentially biasing the analyses. Therefore, multicenter studies with larger samples are needed to validate the findings of this study.

## Conclusions and implications for practice

Based on the findings of the study, parity, social support, family support and level of education are predictors of pregnancy stress. Delivery complications, psychological traumatic event, pregnancy stress and family support are predictors of PBT. The above predictors will aid in the early identification of and intervention in pregnancy stress and PBT. As found in this study, a close relationship exists among pregnancy stress, social support, especially family support, and PBT. Therefore, community health workers and medical personnel should pay attention to the evaluation of pregnancy stress and social support for pregnant women and provide targeted guidance to enhance social support, especially family support for pregnant women as a means of reducing pregnancy stress and preventing PBT.

## Data Availability

The datasets used and/or analyzed during the current study are available from the corresponding author on reasonable request.

## Abbreviations

PBT: Psychological birth trauma
PPTSD: Postpartum post-traumatic stress disorder
PSRS: Pregnancy Stress Rating Scale
City BiTS: City BiTS City Birth Trauma Scale
SSRS: Social Support Rating Scale
Family APGAR: Family Adaptation Partnership Growth Affection and Resolve index
M: Mean
SD: Standard deviation
IQR: Interquartile range
VIF: Variance inflation factor
B: Partial regression coefficient
Beta: Standard regression coefficient
CI: Confidence interval
